# Genetic diversity and structure of one of the most endangered freshwater fish species in Mexico: *Tlaloc hildebrandi* (Miller, 1950) and recognition of its evolutionarily significant units

**DOI:** 10.7717/peerj.11952

**Published:** 2021-08-27

**Authors:** Rosa G. Beltrán-López, Alfonso A. González-Díaz, Miriam Soria-Barreto, Marco Antonio Garduño-Sánchez, Carmela Xochitla-Castrejón, Rocío Rodiles-Hernández, Claudia Patricia Ornelas-García

**Affiliations:** 1 Colección Nacional de Peces, Departamento de Zoología. Instituto de Biología, Ciudad de México, Mexico; 2 Colección de Peces, Departamento de Conservación de la Biodiversidad, El Colegio de la Frontera Sur, San Cristóbal de Las Casas, Chiapas, Mexico; 3 Centro de Investigación de Ciencias Ambientales, Facultad de Ciencias Naturales, Ciudad del Carmen, Campeche, Mexico; 4 Cátedra CONACYT. El Colegio de la Frontera Sur-Unidad San Cristóbal de Las Casas, San Cristobál de Las Casas, Chiapas, Mexico

**Keywords:** Killifish, Conservation biology, *Tlaloc hildebrandi*, Genetic diversity, Endangered species, Mexico, Chiapas, Usumacinta, Grijalva

## Abstract

The endangered Chiapas killifish *Tlaloc hildebrandi* is an endemic freshwater species that lives in four subbasins of the Grijalva and Usumacinta basins, and one of the most geographically restricted species of the Produndulidae family. The species was originally described as endemic to springs in the high limestone plateau in San Cristóbal de Las Casas in the Río Amarillo subbasin (upper Grijalva basin). However, it was recently recorded in the Jataté and Tzaconejá subbasins in the upper Usumacinta basin, thereby expanding its known distribution range. The discovery of these populations is relevant not only for the conservation of the species but also for a better understanding of its evolutionary history. Currently, the scarce populations of *T. hildebrandi*, found in only a few localities in the Grijalva and Usumacinta basins, are fragmented and living under unfavorable conditions. Here, we analyzed three mitochondrial (*mt-atp8&6* and *mt-nd2*) and one nuclear (*nuc-s7*) marker in order to assess the genetic diversity and population structure of *T. hildebrandi*. We found that, in comparison with other endangered freshwater fish species from Mexico, *T. hildebrandi* showed a lower level of genetic diversity (*mt-nd2*: *h* = 0.469, *π* = 0.0009; *mt-atp8&6*: *h* = 0.398, *π* = 0.001; and *nuc-S7*: *h* = 0.433, *π* = 0.001). Moreover, the analyzed populations exhibited a strong genetic structure in accordance with their geographic distribution, and can be placed into three genetic clusters: (1) Amarillo plus Chenhaló in the upper Grijalva basin, (2) Jataté, and (3) Tzaconejá, both in the upper Usumacinta basin. On the basis of our results, we propose the recognition of at least three evolutionarily significant units (ESUs) for the species and the urgent implementation of *ex situ* and *in situ* conservation and management efforts that consider the genetic background of the species.

## Introduction

Profundulidae represents one of the few endemic freshwater families found in the Mesoamerican region. The early divergence and history of this family is reflected by the ancient and complex geohydrological history of its distribution area (Miller, 1966; Myers, 1966; Bussing, 1985; Morcillo et al., 2016). At least two major cladogenic events have been identified for the family: the first gave rise to the lineages leading to the two genera of the family, *Profundulus* and *Tlaloc*, during the early Miocene (26 Mya), and the second was a diversification event during the upper Miocene to early Pliocene (from 5 to 10 Mya) associated with divergences within each of these two genera (Morcillo et al., 2016).

*Tlaloc* is comprised of three species, whose distribution ranges are very restricted, in contrast to their closest relatives in *Profundulus* (Morcillo et al., 2016). *Tlaloc portillorum* is found in the Ulua and Nacaome basins in Honduras, and corresponds to the southernmost *Tlaloc* species. Its two sister species, *T. labialis* and *T. hildebrandi* (Miller, Minckley & Norris, 2005; Morcillo et al., 2016), are both restricted to the upper parts of the Grijalva and Usumacinta basins in Mexico. Miller (1955), in his review of the family, suggested that *T. hildebrandi* is one of the earliest divergent lineages within *Tlaloc*, making this species particularly interesting to our understanding of the evolutionary history of the genus and the family.

**Figure 1:**
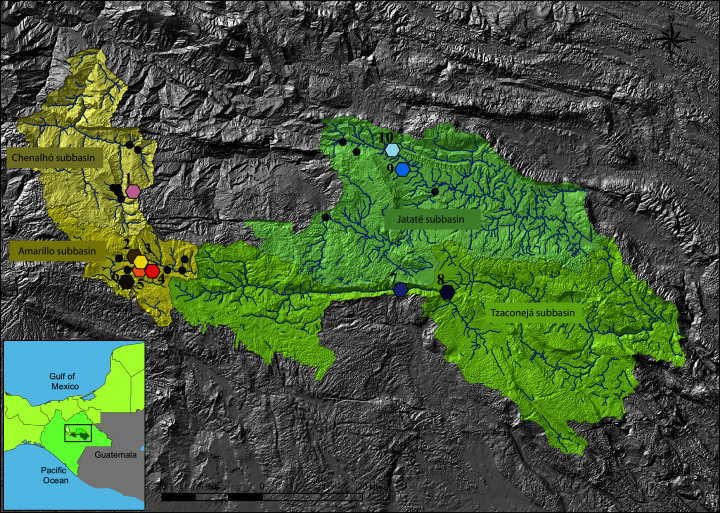
Map showing the geographic location of sampling sites of *T. hildebrandi*. Each one of the four subbasins sampled are shown with different colors (Amarillo and Chenalhó of Grijalva Basin in yellow and, Tzaconeja and Jataté of Usumacinta Basin in green). Black circles represent the distribution records of *T. hildebrandi* reported by Domínguez-Cisneros et al. (2017). Numbers correspond to localities: 1. Chenalhó, 2. Ojo de Agua, 3. Amarillo River, 4. Fogótico Puente, 5. Fogótico Encuentro, 6. Humedales, 7. Naranjal, 8. Tzaconejá, 9. Ocosingo, 10. Las Lajas. Topographic map scale 1:50,000 and Hydrological network map scale 1:50,000 both of Instituto Nacional de Estadística y Geografía (INEGI), availables in https://www.inegi.org.mx/temas/topografia/#Descargas and https://www.inegi.org.mx/temas/hidrologia/#Descargas.

*Tlaloc hildebrandi* is a small cyprinodontid (minimum size: 14.35 mm and maximum size: 110.66 mm standard length; Domínguez-Cisneros et al., 2017) that inhabits lentic and lotic ecosystems in association with rocky bottom habitats and aquatic vegetation. The species is zoobenthivorous: its diet is composed of aquatic insects, crustaceans, amphipods, and mollusks (Velázquez-Velázquez et al., 2007). The species was first described from Laguna María Eugenia in San Cristóbal de Las Casas, a town in the Río Amarillo subbasin (Grijalva basin) in Chiapas, Mexico, in 1950; however, this locality was recorded as dried out (‘disappeared’) a year later (Miller, 1955). It was later found in other ponds close to the type locality and was recognized as a species whose distribution is restricted to freshwater bodies in the high limestone plateau near San Cristóbal de Las Casas, Chiapas. Recently, *T. hildebrandi*, also known as the ‘escamudo de San Cristóbal’ or the Chiapas killifish, was recorded in rivers in the upper part of the Usumacinta basin (Velázquez-Velázquez et al., 2016; Domínguez-Cisneros et al., 2017; Fig. 1). The presence of the species in this basin has implications not only for its conservation but also for our understanding of its evolutionary history.

Unfortunately, the conservation status of *T. hildebrandi* is far from optimum. Due to its restricted distribution and habitat vulnerability, *T. hildebrandi* has been cataloged as Endangered under Mexican legislation (NOM-059-ECOL-2010, SEMARNAT, NOM (Norma Oficial Mexicana), 2010) and also in the International Union for Conservation of Nature (IUCN) Red List of Threatened Species (Schmitter-Soto & Vega-Cendejas, 2019). Populations of the species, which are fragmented and scarce, are currently only known for a few localities in the Grijalva and Usumacinta basins, where they are living under unfavorable conditions (Domínguez-Cisneros et al., 2017), with habitat degradation due to contamination by urban wastewater representing a major threat to the species (Velázquez-Velázquez & Schmitter-Soto, 2004).

Conservation biology is a discipline that aims to guide the management of threatened species, however, often with inadequate information (Soulé & Wilcox, 1980). The IUCN recognizes the need to conserve biodiversity at three levels: ecosystems, species, and genetic diversity (McNeely et al., 1990). Current approaches to biodiversity conservation are largely based on conserving geographic areas, ecosystems, ecological communities, and species, with less attention given to genetic diversity and the species-population continuum (Coates, Byrne & Moritz, 2018). Genetic diversity, however, can be thought of as the foundation of biodiversity, as it facilitates diversification and adaptation to novel and changing environmental conditions, and thus, its conservation is essential to maintain the evolutionary potential of populations (Crandall et al., 2000; Faulks et al., 2016). By characterizing the genetic diversity of a species, we can improve our understanding of the species’ conservation status and better evaluate its extinction risk (Frankham, 2005).

Although still largely overlooked in the practical management of species and in national and international policies (Laikre, 2010; Laikre et al., 2020; Garner, Hoban & Luikart, 2020), the importance of genetic diversity has been elevated in conservation efforts in more recent years. At the last Convention on Biological Diversity (CBD), concern was raised about the dearth of information related to genetic diversity, as a basic element for evolutionary processes, for conservation biology (Laikre et al., 2020). The post-2020 CBD framework proposes that conservation entities should prioritize the maintenance of genetic diversity, with the effective population size (*Ne*), in particular, taken into consideration. This metric is important as it reflects the number of individuals breeding, rather than the total number of individuals in a population. When the *Ne* is small, genetic variation is lost rapidly due to genetic drift and the deleterious effects of inbreeding (Garner, Hoban & Luikart, 2020).

To date, very few genetic studies have been conducted on Profundulidae family, particularly in its most endangered species like *T. hildebrandi*. However, other related cyprinodontiform fishes found in Mesoamerica, primarily belonging to the families Goodeidae and Poeciliidae, have been studied. Genetic studies of species of Goodeidae, including *Zoogoneticus quitzeoensis* (Domínguez-Domínguez et al., 2008), *Neotoca bilineata* (Ornelas-García et al., 2012), the *Allotoca diazi* complex (Corona-Santiago, Doadrio & Domínguez-Domínguez, 2015), and species of *Ilyodon* (Beltrán-López et al., 2017), have allowed the historical and recent events that have shaped their genetic diversity to be identified, and also may be used to guide conservation and management programs. Moreover, Lyons et al. (2019), in their recent study evaluating all known goodeine species, identified 84 evolutionarily significant units (ESUs) for 40 of the species using various lines of evidence (*i.e*., genetics, ecology, and morphology). With respect to the Poeciliidae, *Xiphophorus cortezi* and *Poecilia sulphuraria* have been two of the more well studied species. A low to moderate level of haplotype diversity (*mt-d-loop*: *h* = 0–0.530) was observed for *X. cortezi*, an endemic of the southern Panuco river system (Gutiérrez-Rodríguez et al., 2007), and in *P. sulphuraria*, single nucleotide polymorphism (SNP) analyses of the three known populations of the species revealed a positive correlation between population size and genetic diversity (*i.e*., the largest population had the highest level of genetic diversity, while the smallest had the lowest level) (Brown et al., 2017).

The importance of genetic diversity studies for conservation and the lack of genetic data for the endangered *T. hildebrandi* necessitates a characterization of the species’ genetic diversity, which will improve our knowledge of its current situation and inform conservation efforts. As previously shown, populations with limited ranges can be highly vulnerable to environmental changes, and those impacted by human-induced range reductions often lack genetic diversity information (Brown et al., 2017). We hypothesize that this may be the case in *T. hildebrandi*. The main goal of the present study is to assess the genetic diversity and population structure of *T. hildebrandi* by analyzing three mitochondrial (*mt-atp8&6* and *mt-nd2*) and one nuclear (*nuc-s7*) markers. With this information, we can then establish Evolutionary Significant Units (ESUs) that may prove valuable in the management and conservation of the species.

## Materials and Methods

### Ethical statement

The care and use of animals complied with the SEMARNAT animal welfare laws, guidelines and policies as approved by SEMARNAT-SGA/DGVS/01283/20 collection permit.

### Sample collection and DNA extraction

A total of 33 specimens identified as *T. hildebrandi* on the basis of the taxonomic key of Miller, Minckley & Norris (2005) were collected from 10 localities across the distribution range of the species in the upper Grijalva (Amarillo and Chenalhó subbasins) and the upper Usumacinta (Jataté and Tzaconejá subbasins) basins in Mexico. Samples were collected from all localities in which *T. hildebrandi* is known to occur (Fig. 1; Supplementary Material S1, T1). Fish were captured by electrofishing during 2017 and 2018 with the permission of the local authorities and anesthetized using clove oil. Caudal fins were clipped and preserved in absolute ethanol (>90%) at –20 °C until extraction. Most of the specimens were returned to the water after their fins were clipped. Some individuals were preserved in absolute ethanol (>90%) as vouchers for future morphological analyses. The voucher samples were deposited in the Fish Collection (ECOSC) at ECOSUR in San Cristóbal de Las Casas, Chiapas, Mexico. Measurements of body length by subbasin and voucher catalog numbers are provided in Supplementary Material S1 (T2 and T1 respectively).

DNA was extracted from fin clips as described by Sonnenberg, Nolte & Tautz (2007). Both DNA quality and concentration were measured using a Nanodrop 1000 (Thermo Fisher Scientific, Waltham, MA, USA).

### Mitochondrial and nuclear amplifications

For the DNA analyses, fragments of the mitochondrial NADH dehydrogenase subunit 2 (*mt-nd2*) and ATP synthase 8 and 6 (*mt-atp8&6*) genes were amplified *via* polymerase chain reaction (PCR), as were the first and second introns of the nuclear S7 ribosomal protein gene (*nuc-S7*). Information on the PCR protocols and primers used are provided in Supplementary Material S1 (T3). Reactions were performed in a final volume of 10 μl containing 5.5 μl of nuclease-free water, 2 μl of 5X Mytaq reaction colorless buffer which include the MgCl_2_ and dNTPs standardized, 0.2 μl of each primer (10 μM), 0.1 μl of MyTaq DNA Polymerase (Bioline), and 2 μl (10–100 ng) of DNA template. The PCR amplification conditions used followed Morcillo et al. (2016). The amplicons were purified using ExoSAP-IT (Thermo Fisher Scientific, Waltham, MA, USA) and sequenced by the sequencing facility at the Instituto de Biología of the Universidad Nacional Autónoma de México. Sequences were manually aligned in Mega v10.1.7 (Kumar et al., 2018). Haplotype sequences were deposited in GenBank under the following accession numbers: *mt-nd2*, MW438870–MW438902; *mt-atp8*, MW438903–MW438924; *mt-atp6*, MW438925–MW438946; *nuc-S7*, MW438947–MW438978 (Supplementary Material S1, T2).

### Genetic diversity and haplotype networks

To evaluate the geographic correspondence of the haplotype distribution, network estimations were constructed for each gene fragment using the median-joining algorithm, as implemented in PopArt v1.7 (http://popart.otago.ac.nz).

Genetic diversity parameters, including the number of haplotypes (H) and polymorphic sites (*S*), and nucleotide (π) and haplotype diversity (*h*), were estimated for each gene in Arlequin v3.5.1 (Excoffier & Lischer, 2010). Genetic diversity was assessed for two groupings: (1) groups consider as the two basins in which *T. hildebrandi* is distributed (*i.e*., the upper Grijalva and the upper Usumacinta) and (2) groups considered as the three genetic clusters obtained from the haplotype network analyses, with the two subbasins of the upper Usumacinta basin (Tzaconejá and Jataté subbasins) each comprising a separate group and the Amarillo plus Chenalhó subbasins of the upper Grijalva comprising the third.

### Genetic structure among the populations

To quantify the genetic differences among the sampled populations of *T. hildebrandi*, uncorrected *p-*distances for all genes were calculated considering the same two groupings used to evaluate genetic diversity: (1) groups considered as the two basins in which *T. hildebrandi* is distributed (upper Grijalva and upper Usumacinta) and (2) the three major groups indicated by the haplotype networks, which are the Amarillo plus Chenalhó subbasins, the Tzaconejá subbasin, and the Jataté subbasin.

Genetic differentiation among the sampled populations was estimated with paired test fixation indices (*Φ*_*ST*_) for each gene, with Bonferroni correction (Rice, 1989). These analyses were implemented according to the same groupings described above.

To analyze the genetic structure of the populations of *T. hildebrandi*, analyses of molecular variance (AMOVAs) were conducted using Arlequin v3.5.1.3 (Excoffier & Lischer, 2010) at two hierarchical levels, using the same groupings as in the previous analyses. Components of the fixation indices, *Φ*_*CT*_, *Φ*_*ST*_, and *Φ*_*SC*_, were also calculated using Arlequin v3.5.1.3.

### Gene flow between basins

To estimate the level and direction of historical gene flow between the hydrological basins of Grijalva and Usumacinta, migration rates for *T. hildebrandi* were computed using MIGRATE-N 3.6 (Beerli, 2009). MCMC simulations were performed as follows: two long chains and 12 short parallel chains with initial temperatures of 1.0, 1.5, 3.0, and 100,000.0, and a static heating scheme. Final MCMC searches used 10,000,000 steps in 50-step increments, discarding the first 1,000,000 as burn-in. Initial uniform priors were Θ (0.0–0.1) and M (0.0–20,000.0).

## Results

### Samples and sequence data

We successfully amplified 33 sequences of *mt-nd2* (764 bp), 22 of *mt-atp8&6* (788 bp), and 32 of *nuc-S7* (819 bp), from the samples collected from 10 localities across the distribution range of *T. hildebrandi* in the upper Grijalva and upper Usumacinta basins (Supplementary Material S1, T2 and Fig. 1). For *mt-nd2*, seven sites were polymorphic, of which two were parsimony informative, and five were singletons. For *mt-atp8&6*, four sites were polymorphic, of which two were parsimony informative, and two were singletons. Finally, for *nuc-S7*, four sites were polymorphic, of which one was parsimony informative, and three were singletons.

### Genetic diversity and haplotype networks

Genetic diversity, calculated considering all populations as a single group, was highest for *mt-nd2* (*h* = 0.469; *π* = 0.0009), followed by *nuc-S7* (*h* = 0.433; *π* = 0.001) and *mt-atp8&6* (*h* = 0.398; *π* = 0.001). When genetic diversity was calculated by basin (*i.e*., upper Grijalva *vs* upper Usumacinta), the upper Usumacinta (*i.e*., Tzaconejá and Jataté subbasins) showed a higher level of genetic diversity for all loci, with *mt-atp8&6* (*h* = 0.785; *π* = 0.001) having the highest level, followed by *nuc-S7* (*h* = 0.530 *π* = 0.001) and *mt-nd2* (*h* = 0.525; *π* = 0.0012). The low level of genetic diversity in the upper Grijalva basin (*i.e*., Amarillo and Chenalhó subbasins) is mainly reflected by the lack of diversity found for *mt-atp8&6* and *nuc-S7* (Table 1). Finally, when genetic diversity was calculated by subbasin for the upper Usumacinta (*i.e*., Tzaconejá *vs* Jataté), Jataté showed a higher level of genetic diversity for the three genes (*mt-nd2*: *h* = 0.833, *π* = 0.001; *mt-atp8&6*: *h* = 0.7, *π* = 0.001; and *nuc-S7*: *h* = 0.666, *π* = 0.001; Table 1).

**Table 1:** Genetic diversity for three groupings, 1) considering three groups according to the Rivers where *T. hildebrandi* is distributed, 2) considering two groups according to geographic distribution (upper Grijalva and upper Usumacinta), and 3) considering all populations as a single group. N, sample size, S, polymorphic sites, H, number of haplotypes, π, nucleotide diversity *h*, haplotype diversity.

	N	S	H	*π*	*h*
***mt-nd2***					
1) Amarillo + Chenalhó subbasin	20	4	4	0.0007 ± 0.000	0.431 ± 0.126
Tzaconeja subbasin	9	0	1	0.000 ± 0.000	0.000 ± 0.000
Jataté subbasin	4	3	3	0.001 ± 0.001	0.833 ± 0.222
2) upper Grijalva	20	4	4	0.0007 ± 0.000	0.431 ± 0.126
upper Usumacinta	13	4	4	0.0012 ± 0.001	0.525 ± 0.152
3) A single group	33	7	7	0.0009 ± 0.000	0.469 ± 0.104
***mt-atp8&6***					
1) Amarillo + Chenalhó subbasin	14	0	1	0.000 ± 0.000	0.000 ± 0.000
Tzaconeja subbasin	3	0	1	0.000 ± 0.000	0.000 ± 0.000
Jataté subbasin	5	3	3	0.001 ± 0.001	0.700 ± 0.218
2) upper Grijalva	14	0	1	0.000 ± 0.000	0.000 ± 0.000
upper Usumacinta	8	4	4	0.001 ± 0.001	0.785 ± 0.112
3) A single group	22	4	4	0.001 ± 0.000	0.398 ± 0.121
***nuc-S7***					
1) Amarillo + Chenalhó subbasin	20	0	1	0.000 ± 0.000	0.000 ± 0.000
Tzaconeja subbasin	9	4	3	0.001 ± 0.001	0.416 ± 0.190
Jataté subbasin	3	2	2	0.001 ± 0.001	0.666 ± 0.314
2) upper Grijalva	20	0	1	0.000 ± 0.000	0.000 ± 0.000
upper Usumacinta	12	4	3	0.001 ± 0.001	0.530 ± 0.135
3) A single group	32	5	3	0.001 ± 0.000	0.433 ± 0.081

**Figure 2:**
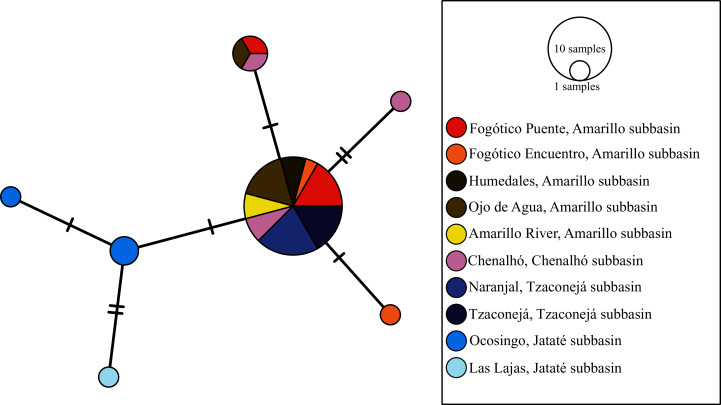
Haplotype network of *mt-nd2* for all sampled populations of *T. hildebrandi*. The size of the circles represents the relative frequency of sequences belonging to a particular haplotype. Marks along the network branches indicate the number of mutation steps. Each color represents a different population of the four subbasins sampled.

**Figure 3:**
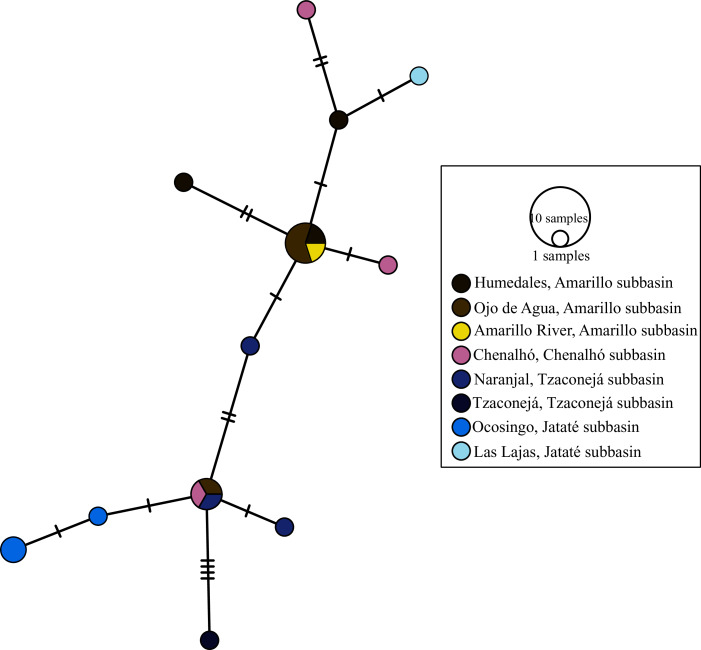
Haplotype network of *mt-atp8&6* for all sampled populations of *T. hildebrandi*. The size of the circles represents the relative frequency of sequences belonging to a particular haplotype. Marks along the network branches indicate the number of mutation steps. Each color represents a different population of the four subbasins sampled.

The haplotype networks of the mitochondrial markers were very similar. The haplotype network of *mt-nd2* showed seven haplotypes, with a visible structure among subbasins. The most common haplotype for *mt-nd2* and for *mt-atp8&6* (24 sequences for *mt-nd2* and 17 sequences for *mt-atp8&6*) was recovered from samples from the Amarillo subbasin (upper Grijalva) in San Cristóbal de Las Casas, Chiapas (*i.e*., Fogótico Puente, Fogótico Encuentro, Humedales, and Ojo de Agua, and in the case of *mt-nd2*, Río Amarillo (sequences of *mt-atp8&6* were not amplified for this last population)), those from the Chenalhó subbasin (upper Grijalva), and some from the Tzaconejá subbasin of the upper Usumacinta (Naranjal and Tzaconejá populations). One mutation step separated the most common haplotype of *mt-nd2* from the rest of the haplotypes, except for one haplotype from the Chenalhó subbasin, which was separated by two mutation steps. The peripheral haplotypes, separated by one mutation step, included samples from the Amarillo and Chenalhó subbasins and those from the Jataté subbasin (upper Usumacinta). The Ocosingo and Las Lajas populations (upper Usumacinta), which did not have any shared haplotypes, were separated by two mutation steps (Fig. 2). The most common haplotype for *mt-atp8&6* was separated by one mutation step from the one in the Jataté subbasin (upper Usumacinta). Like for *mt-nd2*, the populations of Ocosingo and Las Lajas were separated by two mutation steps, and did not share any haplotypes (Fig. 3).

**Figure 4:**
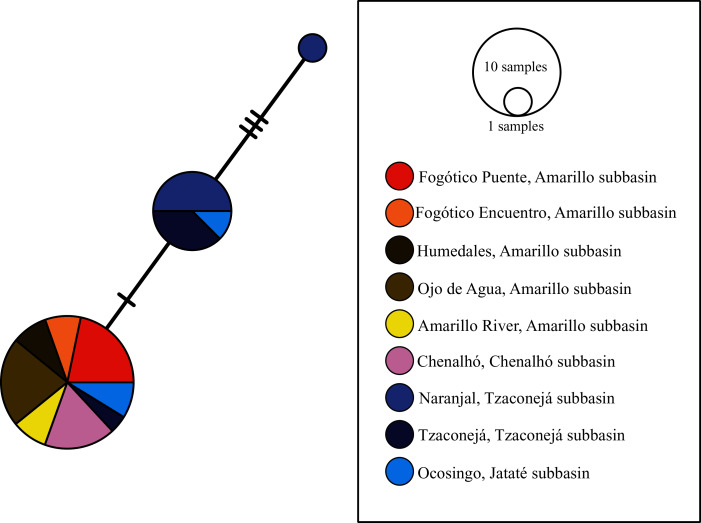
Haplotype network of *nuc-S7* for all sampled populations of *T. hildebrandi*. The size of the circles represents the relative frequency of sequences belonging to a particular haplotype. Marks along the network branches indicate the number of mutation steps. Each color represents a different population of the four subbasins sampled.

The haplotype network of *nuc-S7* showed three haplotypes. The most common haplotype (23 sequences) was found in samples from the Amarillo subbasin of the upper Grijalva (*i.e*., Fogótico Puente, Fogótico Encuentro, Humedales, Ojo de Agua, and Rio Amarillo), those from the Chenalhó subbasin, two from Ocosingo (Jataté subbasin) and one from the Tzaconejá subbasin. This haplotype was separated by one mutation step from the one found in the El Naranjal and Tzaconejá (Tzaconejá subbasin) and Ocosingo (Jataté subbasin) populations in the upper Usumacinta basin. Additionally, an exclusive haplotype separated by three mutation steps from the nearest haplotype was found in the El Naranjal population (Fig. 4).

To summarize, the Jataté subbasin showed exclusive mitochondrial haplotypes not shared with the Tzaconejá subbasin, despite both being in the upper Usumacinta. Moreover, the populations from the Tzaconejá subbasin shared haplotypes with those from the Amarillo and Chenalhó subbasins of the upper Grijalva (Figs. 2 and 3). By contrast, for the *nuc-S7* gene, shared haplotypes were found between the Tzaconejá and Jataté subbasins of the upper Usumacinta, and only one shared haplotype was found between one of the Usumacinta subbasins, in this case Jataté, and the Amarillo and Chenalhó subbasins (Fig. 4).

### Genetic structure among populations

The genetic distance (uncorrected *p*-distances) between the two basins (upper Grijalva *vs* upper Usumacinta) was 0.1% for *mt-nd2* and 0.2% for *mt-atp8&6* and *nuc-S7* (Table 2). When the two Usumacinta subbasins were considered as independent groups, the lowest genetic distances for *mt-nd2* were observed between the Tzaconejá and the Amarillo + Chenalhó subbasins (0%), whereas the highest distance was observed between the Jataté and Amarillo + Chenalhó subbasins (0.3%). Results for *mt-atp8&6* were the same as for *mt-nd2*, except that a relatively high genetic distance was also found between the Jataté and Tzaconejá subbasins (0.3%). By contrast, for *nuc-S7*, the lowest genetic distance was observed between the Jataté and the Amarillo + Chenalhó subbasins (0.1%), and the highest was between the Tzaconejá and the Amarillo + Chenalhó subbasins (0.2%), though the Tzaconejá and Jataté subbasins also showed a distance of 0.2% (Table 2).

**Table 2:** Below of diagonal are shown uncorrected *p* genetic distances in percentage within (bold numbers) and between groups, and above of diagonal and italics letters are shown genetic differentiation using pairwise *Φ*_ST_. In bold are significant values after Bonferroni correction (*p* < 0.05). Both analyses implemented with the three genes *ND2*, *ATP6* and *S7*, the used arrangement was according to (1) three rivers, and (2) two regions (upper Grijalva and upper Usumacinta).

***mt-nd2***			
1) Three groups	Amarillo River	Tzaconeja River	Jataté River
Amarillo + Chenalhó subbasins	**0.1%**	*−0.005*	0.612**
Tzaconeja subbasin	0%	**0%**	0.731**
Jataté subbasin	0.3%	0.2%	**0.2%**
2) Two Regions	upper Grijalva	upper Usumacinta	
upper Grijalva	**0.1%**	0.117**	
upper Usumacinta	0.1%	**0.1%**	
***mt-atp8&6***			
1) Three groups	Amarillo River	Tzaconeja River	Jataté River
Amarillo + Chenalhó subbasins	**0.0%**	*0.000*	0.809**
Tzaconeja subbasin	0.0%	**0.0%**	0.560**
Jataté subbasin	0.3%	0.3%	**0.2%**
2) Two Regions	upper Grijalva	upper Usumacinta	
upper Grijalva	**0.0%**	0.480**	
upper Usumacinta	0.2%	**0.2%**	
***nuc-S7***			
1) Three groups	Amarillo River	Tzaconeja River	Jataté River
Amarillo + Chenalhó subbasins	**0%**	0.823**	*0.609*
Tzaconeja subbasin	0.2%	**0.1%**	*0.231*
Jataté subbasin	0.1%	0.2%	**0.1%**
2) Two Regions	upper Grijalva	upper Usumacinta	
upper Grijalva	**0%**	0.692**	
upper Usumacinta	0.2%	**0.1%**	

**Note:**

** Significant values after Bonferroni correction (*p* < 0.05).

Pairwise *Φ*_ST_ comparisons between the two basins (upper Grijalva *vs* upper Usumacinta) resulted in *Φ*_ST_ values of 0.117 for *mt-nd2*, 0.480 for *mt-atp8&6*, and 0.692 for *nuc-S7*, all of which were significant. However, pairwise comparisons among the three main groups identified by the haplotype analyses showed a lack of genetic differentiation between Tzaconejá and the Amarillo + Chenalhó subbasins for *mt-nd2* (*Φ*_ST_ = 0), though significant differentiation between Jataté and the Amarillo + Chenalhó subbasins, and also between Jataté and Tzaconejá (*Φ*_*ST*_ = 0.612 and 0.731, respectively; *p* < 0.05). Significant differentiation between the same subbasins for *mt-nd2* was observed for *mt-atp8&6*, though with higher *Φ*_ST_ values (*Φ*_ST_ = 0.809 and, 0.560 respectively, between Jataté and the Amarillo + Chenalhó subbasins and between Jataté and Tzaconejá). Finally, for *nuc-S7*, significant differentiation was only observed between Tzaconejá and the Amarillo + Chenalhó subbasins (*Φ*_ST_ = 0.823); moderate but non-significant differences were found between Jataté and the Amarillo + Chenalhó subbasins (*Φ*_ST_ = 0.609, Table 2).

In the AMOVA with *mt-nd2*, no significant differences were observed between groups when samples were grouped by basin (upper Usumacinta *vs* upper Grijalva); however, differences between populations and within populations were significant (Table 3). In the AMOVA with *mt-atp8&6*, variation between basins was considerably higher and significant at 35.60%, while the amount of variance between populations within groups and within populations was lower but significant (*Φ*_SC_ = 0.84 and *Φ*_ST_: 0.89, Table 3). Finally, the analysis with *nuc-S7* showed that differences between the two basins accounted for 62.17% of the variance (*Φ*_CT_ = 0.62, *Φ*_SC_ = 0.29, and *Φ*_ST_: 0.73), with *Φ*_ST_ as the only significant factor. When the samples were grouped according to the three groups indicated by the haplotype analyses, the percentage of variation among groups was as follows: for *mt-nd2*, 40.02% (*Φ*_CT_ = −0.40, *Φ*_SC_ = −0.11, and *Φ*_ST_: 0.47); for *mt-atp8&6*, 66.70% (*Φ*_CT_ = 0.66, *Φ*_SC_ = 0.72 and *Φ*_ST_: 0.90); and for *nuc-*S7, 76.94% (*Φ*_CT_ = 0.76, *Φ*_SC_ = −0.18 and *Φ*_ST_: 0.72), and only the *Φ*_ST_ value was significant for all three markers (Table 3).

**Table 3:** Analyses of molecular variance for two grouping schemes and by gene.

Testing assumptions	Source of variation	% of variance	Fixation index
***mt-nd2***			
Testing assumptions	Source of variation	% of variance	Fixation index
1) Amarrilo + Chenalhó subbasins/Tzaconeja subbasin/Jataté subbasin	Among groups Among populations within groups Within populations Total	40.02 7.15 52.83 100	*Φ*_CT_: 0.40 *Φ*_SC_: −0.11 *Φ*_ST_: 0.47*
2) upper Grijalva/upper Usumacinta	Among groups Among populations within groups Within populations Total	−29.12 67.64 61.48 100	*Φ*_CT_: −0.29 *Φ*_SC_: 0.52* Φ_ST_: 0.38*
***mt-atp8&6***			
1) Amarrilo + Chenalhó subbasins/Tzaconeja subbasin/Jataté subbasin	Among groups Among populations within groups Within populations Total	66.70 24.23 9.07 100	*Φ*_CT_: 0.66 *Φ*_SC_: 0.72 *Φ*_ST_: 0.90*
2) upper Grijalva/upper Usumacinta	Among groups Among populations within groups Within populations Total	35.60 54.17 10.23 100	*Φ*_CT_: 0.35* Φ_SC_: 0.84* Φ_ST_: 0.89*
***nuc-S7***			
1) Amarrilo + Chenalhó subbasins/Tzaconeja subbasin/Jataté subbasin	Among groups Among populations within groups Within populations Total	76.94 −4.27 27.34 100	*Φ*_CT_: 0.76 *Φ*_SC_: −0.18 *Φ*_ST_: 0.72*
2) upper Grijalva/upper Usumacinta	Among groups Among populations within groups Within populations Total	62.17 11.21 26.62 100	*Φ*_CT_: 0.62 *Φ*_SC_: 0.29 *Φ*_ST_: 0.73*

**Note:**

* Significant values

### Gene flow between basins

The lowest migration rates were observed for *mt-nd2* (_m_Grijalva–Usumacinta = 0.0036 and _m_Usumacinta–Grijalva = 0.0028). Migration rates for *mt-atp8&6* (_m_Grijalva–Usumacinta = 0.0162 and _m_Usumacinta–Grijalva = 0.0147) were lower than those for *nuc-S7* (_m_Grijalva–Usumacinta = 0.1205 and _m_Usumacinta–Grijalva = 0.1511). The results for the mitochondrial genes indicate a greater magnitude and direction of migrants from Grijalva to Usumacinta than from the other direction. By contrast, the rates for *nuc-S7* indicate greater migration from Usumacinta to Grijalva (Supplementary Material S1, T4).

## Discussion

The geographic distribution of *T. hildebrandi* was long thought to be restricted to only the Amarillo subbasin, specifically in San Cristóbal de las Casas, Chiapas; however, in 2016, Velázquez-Velázquez et al. (2016) expanded the geographic range of the species to include the Ocosingo River in the upper Usumacinta basin. This distribution pattern suggested that populations of *T. hildebrandi* may exhibit some level of genetic diversity and structure, however, until now, no genetic studies had been carried out to evaluate this hypothesis. The present study is the first to characterize the genetic diversity of all known populations of *T. hildebrandi*, and to propose guidelines for the conservation and management of the species based on its genetic diversity.

### Genetic diversity

Notably, our results highlight the generally low level of genetic diversity of current populations of *T. hildebrandi*, at least according to the three gene fragments analyzed: *mt-nd2* (*h* = 0.469; *π* = 0.0009), *mt-atp8&6* (*h* = 0.398; *π* = 0.001), and *nuc-S7* (*h* = 0.433; *π* = 0.001). The genetic diversity values obtained for *T. hildebrandi* are considerably lower than those reported for some other endangered Cyprinodontiformes in Mexico. For instance, in the viviparous goodeine *Z. quitzeoensis*, which still has at least 10 population across the Lerma-Chapala basin, high levels of haplotype and nucleotide diversity (*mt-cytb*: *h* = 0.714–1.00, *π* = 0.0015–0.0049) were observed, and on the basis of these results, seven operational conservation units (OCUs) were identified for the species (Domínguez-Domínguez et al., 2008). Similarly, in another goodeine, *N. bilineata*, whose populations in the middle Lerma-Chapala basin have declined across almost 70% of its original distribution range, haplotype and nucleotide diversity of mitochondrial markers (*i.e*., *mt-cytb*: *h* = 0.971, *π* = 0.006; Ornelas-García et al., 2012) were considerably higher than those found in our study. Likewise, in the goodeine *A. diazi* species complex, high genetic diversity was observed for both *A. diazi* (*mt-cytb*: *h* = 0.78, *π* = 0.003) and *A. meeki* (*mt-cytb*: *h* = 0.52, *π* = 0.0007) (Corona-Santiago, Doadrio & Domínguez-Domínguez, 2015).

However, similar levels of genetic diversity have been reported for some other freshwater fish species in Mexico. For instance, in the critically endangered butterfly splitfin *Ameca splendens*, moderate values of genetic diversity (*He* ranging from 0.34 to 0.94) were found (Hamill et al., 2007). Likewise, for *X. cortezi*, a species belonging to the Poeciliidae, the genetic diversity of *mt-d-loop* ranged from *h* = 0.00 to 0.530 (Gutiérrez-Rodríguez et al., 2007). Several authors have argued that such patterns of low genetic diversity are most likely the consequence of small population sizes, which result due to the effects of genetic drift and inbreeding (Nei, Maruyama & Chakraborty, 1975; Wishard et al., 1984; Gutiérrez-Rodríguez et al., 2007). In a SNP marker analysis of another poeciliid species, the endangered *P. sulphuraria*, nucleotide diversity values (from *π* = 0.0012 to 0.0014) were similar to those founded for *T. hildebrandi*; also, in this case, the population with the largest size had the highest level of genetic diversity (Brown et al., 2017). We explored a possible relationship between genetic diversity and geographic area of distribution (as *a proxy* for population size) for *T. hildebrandi* and some of aforementioned species. Based on estimates using Google Earth, the goodeines *Z. quitzeoensis* (7,000 km^2^) and *N. bilineata* (2,600 km^2^) have the largest geographic distributions, followed by the poeciliid *X. cortezi* (2,000 km^2^), *T. hildebrandi* (1,500 km^2^), and *P. sulphuria* (0.24 km^2^). The poeciliids, which appear to be more geographically restricted, all present a similar low level of genetic diversity compared with the goodeines, consistent with the idea that genetic diversity is correlated with distribution area. Although there are no specific studies on population size in *T. hildebrandi*, the low number of individuals reported in previous ecological studies (Domínguez-Cisneros et al., 2017) suggests a small *Ne*, which has been shown to account for low levels of genetic diversity in other species (Gutiérrez-Rodríguez et al., 2007; Brown et al., 2017).

In this regard, *T. hildebrandi* presents a great conservation challenge. Among the freshwater fish species in Mexico, it has one of the lowest levels of genetic diversity reported so far, making its extinction risk high and its conservation urgent (Frankham, 2005). Moreover, little is known about the demography of current populations of the species, thus, conservation and management programs need to consider both the ecological and the genetic (diversity and structure) data in order to protect the distinctiveness of populations (Crandall et al., 2000). In the case of *T. hildebrandi*, the strong, although not significant, genetic structure observed on the basis of the three analyzed genes (mitochondrial *mt-nd2* and *mt-atp8&6* and *nuc-S7*) suggests the distinctiveness of populations within the two upper Usumacinta subbasins and the upper Grijalva basin (see Tables 2 and 3). Additionally, the haplotype networks of these genes showed a similar pattern in which a common haplotype was present in all populations from the upper Grijalva and some populations from one of the two upper Usumacinta subbasins (see Figs. 2–4). These specific data should, therefore, be considered in the conservation and management of the species in the distinct subbasins.

### Phylogeographic pattern

Although phylogeographic studies of other species distributed in the Grijalva and Usumacinta basins have been performed (*e.g*., in Characiforms; Ornelas-García, Maya-Bernal & Rodiles-Hernández, 2019), *T. hildebrandi* is unique in that its populations are distributed across four subbasins in only the upper part of the two basins, providing an opportunity to explore phylogeographic patterns not previously described. According to the distribution of some freshwater fishes in Middle America, specifically cichlids and poeciliids, the upper Grijalva and the upper Usumacinta represent important areas of endemism (Elías et al., 2020). Both regions contain not only a large number of endemic species but also unique assemblages and a molecular diversity not shared with the lower portion of the same river network.

In this context, our results showing discordance between mitochondrial and nuclear markers present an interesting phylogeographic pattern that could be due to two alternative scenarios: recent secondary contact or incomplete lineage sorting. In the first, recent secondary contact could have occurred between the Tzaconejá subbasin (upper Usumacinta) and the Amarillo and Chenalhó subbasins (upper Grijalva), given their geographic proximity and the geographic features of the region. These features, possibly related to geological events, such as reversal flow and collapse of the stream bed, could have allowed underground connectivity among the subbasins (Rosen, 1979).

Indeed, it has been shown that some surface water stream beds in the geographic region under study partially collapsed in the past and formed subterranean passages that permitted underground connectivity among rivers (Rosen, 1970; Rosen, 1979). Geological evidence suggests that the upper parts of Usumacinta and Grijalva could have been connected from the late Jurassic to the upper Cretaceous. These include a series of geological events that caused the uplift of several mountains in the region (Padilla, 2007), folds and faults that occurred during the Miocene (Carfantan, 1981), and the formation of karst and extensive areas comprised of limestone rocks (Lugo, 1990). These events likely shaped the connections between the two basins, which could have impacted the migration and connectivity of populations of *T. hildebrandi*. According to our Migrate analysis, migration occurred in both directions between the two basins, supporting their historical geographic connection. The genetic differentiation observed in the AMOVAs and the patterns observed in the haplotype networks (see Table 3; Figs. 2 and 3) can be explained in light of the observed genetic diversity of the current populations of *T. hildebrandi* and also the relatively low level of gene flow observed between the two basins. Moreover, some studies have reported a high level of hydrological permeability between basins (see Morán-Zenteno et al., 2000), making a hydrological connection between some populations plausible, for instance between the Río Fogótico population in the Amarillo subbasin and the El Naranjal population in the Tzaconejá subbasin (separated by approximately 6.95 km).

Previous studies of ichthyofaunal assemblages distributed in an area between the upper parts of the Grijalva and Usumacinta basins and Río Comitán and Lake Montebello showed that this area is composed of species found in both basins (*i.e*., *Poeciliopsis hnilickai*, *Chiapaheros grammodes*, *Vieja hartwegi*, and *Xiphophorus alvarezi*), indicating its colonization by species from both regions (Elías et al., 2020). The same pattern of historical river capture or subterraneous connectivity could explain the presence of shared haplotypes (although at low frequencies) between the upper parts of the Grijalva and Usumacinta basins, and the observed level of gene flow between regions; however, more evidence is required to test this hypothesis.

The alternative explanation for the shared haplotypes is incomplete lineage sorting associated with the lower mutation rate of *nuc-S7* compared with the mitochondrial genes. As a result, the populations in the Jataté and the Amarillo and Chenalhó subbasins appear closely related in the *nuc-S7* haplotype network. Similar findings have been observed for other freshwater fishes (Beltrán-López et al., 2017, Beltrán-López et al., 2018; Betancourt-Resendes, Pérez-Rodríguez & Domínguez-Domínguez, 2018, Betancourt-Resendes et al., 2019). The presence of both nuclear and mitochondrial shared haplotypes between the upper Grijalva and upper Usumacinta basins would more robustly support a connection between them. In order to distinguish between the two proposed scenarios, we suggest the inclusion of markers such as SNPs in future analyses.

With respect to the genetic structure of the basins, in our AMOVA analyses (see Table 3), the greatest variance was observed when groups were considered as the three genetic clusters identified in the haplotype network analysis (*i.e*., the Tzaconejá subbasin, the Jataté subbasin, both in the upper Usumacinta, and the Amarillo plus Chenalhó subbasins in the upper Grijalva). Likewise, pairwise *Φ*_ST_ values were higher when comparing the three groups as opposed to the two basins (Table 2). Taken together, these findings indicate a potential substructure within the upper Usumacinta basin that could be related to the high level of endemism of freshwater fishes in the region (Elías et al., 2020) or to the current configuration of the Tzaconejá and Jataté subbasins, which has resulted in their isolation from one another, making the interchange of individuals impossible.

Given the limited distribution of the species, genetic structuration among populations of *T. hildebrandi* was expected. We hypothesize that other freshwater fish species distributed in the same region will also exhibit a similar phylogeographic pattern. Although there have been some phylogeographic studies of fishes distributed in the upper Usumacinta, including species of the genera *Rhamdia, Cichlidae*, and *Poeciliidae*, the low sample numbers collected for the region precluded an analysis of genetic structure (Perdices et al., 2002; Elías et al., 2020). A strong genetic structure, however, has been reported for other species as *Z. quitzeoensis* and *N. bilineata*, which have a restricted distribution, mainly due to habitat fragmentation, but in another geographic region of the country (central Mexico) (Domínguez-Domínguez et al., 2008; Ornelas-García et al., 2012).

### Implications for conservation

*Tlaloc hildebrandi* is cataloged as an endangered species by both the IUCN (Schmitter-Soto & Vega-Cendejas, 2019) and NOM-059-ECOL-2010, SEMARNAT-2010 (NOM-059-ECOL-2010, SEMARNAT-2010). In combination with previous studies on the biological and ecological features of *T. hildebrandi*, genetic studies can provide a better understanding of the current conservation status of the species. This first study of the genetic diversity of the species may prove useful for establishing a management program for this high-risk species (Faulks et al., 2016). The low level of genetic diversity observed for *T. hildebrandi* could be due to two factors: (1) a restricted geographic distribution (Brown et al., 2017) and (2) a small population size, which may be a consequence of human-mediated activities (Gutiérrez-Rodríguez et al., 2007). Unfortunately, at present, little is known about the effective population size (*i.e*., the *Ne*) of the species, despite its crucial importance for conservation efforts. In recent works demonstrating the wider than previously thought distribution of *T. hildebrandi*, very few specimens were collected from the different sampling localities with the exception of Chenalhó (Domínguez-Cisneros et al., 2017). The water quality at this locality is higher than at the others (A. González-Díaz, 2021, personal communication), which may contribute to the potentially larger population size of *T. hildebrandi* at this site.

In recent decades, there has often been a lack of connection between the genetic information of a species and the conservation policies enacted for its protection (Laikre, 2010; Santamaría & Méndez, 2012; Garner, Hoban & Luikart, 2020). This represents an obstacle to the effective conservation of species, considering that inbreeding, genetic drift, and loss of genetic diversity are widely recognized processes that reduce the viability of populations and increase their extinction risk (Frankham, 2005). These factors, together with threats associated with habitat deterioration, could promote demographic stochasticity (Lande, 1981). Over the last few years, however, the importance of knowing the genetic diversity of species for conservation has increased, and more conservation strategies are taking it into consideration (Garner, Hoban & Luikart, 2020; Laikre et al., 2020).

*Tlaloc hildebrandi* represents one of the most geographically restricted species of the family Profundulidae (Miller, Minckley & Norris, 2005). Moreover, in recent years, its area of distribution has undergone rapid environmental deterioration. Loss and degradation of freshwater habitats in the region is caused mainly by water pollution, agricultural and urban development. As a result, populations of *T. hildebrandi* have become isolated and, with the loss of connectivity, have reduced in size (unpublished data). Another threat to the species is the introduction of exotic species. Parasites of the exotic species *Cyprinus carpio* have been shown to negatively affect larvae and juveniles of *T. hildebrandi* (Velázquez-Velázquez, González-Solís & Salgado-Maldonado, 2011).

Despite the very low genetic diversity observed in *T. hildebrandi*, our results indicate a strong genetic structure among the analyzed subbasins (*i.e*., Jataté, Tzaconejá, and Amarillo and Chenalhó; see Tables 2 and 3). In these four subbasin, exclusive haplotypes that can inform on the evolutionary history of the species were found, which may prove relevant for conservation efforts. We suggest the establishment of an immediate management program for the species that considers at least three ESUs, each representing a unique gene pool: (1) the Jataté and (2) the Tzaconejá subbasins, both in the upper Usumacinta, and (3) the Amarillo and Chenalhó subbasins in the upper Grijalva.

The handful of successful cases of preserving endangered species in Mexico have included both *in situ* and *ex situ* conservation strategies. In particular, *in situ* conservation initiatives for freshwater fishes of Mexico have been limited to a couple of Cyprinodontiformes, including the endemic *Cyprinodon julimes* (De la Maza-Benignos & Vela-Valladares, 2009), whose habitat was declared a Ramsar site (https://rsis.ramsar.org/ris/2201). One of the most significant *in situ* conservation projects involves the reintroduction of *Zoogoneticus tequila* (Goodeidae) (once considered extinct in the wild) into the Teuchitlán River in Jalisco (Domínguez-Domínguez et al., 2018) using *ex situ* reproductive stocks. Due to the low genetic diversity of *T. hildebrandi*, it is necessary to establish *ex situ* reproductive stocks representing each of the proposed ESUs as a first step in the conservation of its genetic diversity. This, along with *in situ* strategies, will allow for the persistence of the species.

## Conclusions

Genetic diversity plays an important role in the conservation management of populations of endangered species. *Tlaloc hildebrandi* represents an endangered species with a highly restricted geographic distribution whose habitats are rapidly deteriorating. Main threats to the species are population isolation, environmental contamination, habitat degradation, and exotic species introductions. Given the genetic structure exhibited by its populations in accordance with their geographic distribution, we suggest the recognition of at least three ESUs. We also propose urgent implementation of *ex situ* and *in situ* conservation strategies, including the maintenance and breeding of select populations with relatively higher levels of genetic diversity and abundance in fish tanks (an *ex situ* strategy) and the establishment of protected areas in its distribution range, habitat restoration, and the reintroduction of the species to some rivers (*in situ* strategies). Finally, we recommend the promotion of societal activities related to environmental education, and improved communication between the scientific community and the authorities and decision makers who enact policies at the local and state levels.

## Supplemental Information

10.7717/peerj.11952/supp-1 Supplemental Information 1 Supplemental Tables.

10.7717/peerj.11952/supp-2 Supplemental Information 2 ND2 Fasta File.

10.7717/peerj.11952/supp-3 Supplemental Information 3 S7 Fasta File.

10.7717/peerj.11952/supp-4 Supplemental Information 4 mtATP6 Fasta File.

10.7717/peerj.11952/supp-5 Supplemental Information 5 mtATP8 Fasta File.

## Additional Information and Declarations

### Field Study Permissions

The following information was supplied relating to field study approvals (*i.e*., approving body and any reference numbers): The care and use of animals complied with the SEMARNAT animal welfare laws, guidelines and policies as approved by SEMARNAT-SGA/DGVS/01283/20.

### Data Availability

The following information was supplied regarding data availability: Our sequences were deposited to NCBI GenBank, thus, no further raw data was generated. The GenBank accession numbers are, *mt-nd2*: MW438870–MW438902; *mt-atp8*: MW438903–MW438924; *mt-atp6*: MW438925–MW438946; *nuc-S7*: MW438947–MW438978.
